# Hospital Ships

**Published:** 1896-09-26

**Authors:** 


### HOSPITAL SHIPS.

The *Lancet* of April 4th, 1896, contains a paper by Dr. P. M. Braidwood, upon "Hospital Accommodation by the Use of Ships," in which the author reiterates and expands the ideas propounded by him about twelve years ago as to the desirability of constructing special steamships for hospital purposes. He would have such a ship to accompany each fleet of war, and in addition to this he would have a naval ambulance association, analogous to the Red Cross societies for the aid of the wounded of armies in time of war. It is not quite clear whether his hospital ships are to be constructed, fitted, and controlled by the Navy Department or by his Naval Ambulance Association, but he appears to favour the latter plan. It seems probable that in the coming years the Government will have sufficient need of hospitals of 250 beds each to warrant the construction and fitting up in the best possible way of at least two such ships, instead of relying upon hiring passenger steamers in all emergencies; but it is by no means clear that these hospital ships will be required exclusively, or even mainly, for the service of the navy, and it is, to say the least, extremely doubtful whether they should be in the hands of a volunteer Red Cross association. The chief use of such ships will be the transport of sick and wounded soldiers, although for the greater part of the time they would no doubt be used as floating localised hospitals. It would be a rather peculiar naval battle which would be suitable for Dr. Braidwood's plan of work for his "Naval Ambulance Association." He defines its aim to be "to succour all during and after the battle, without regard to nationality, and to proceed with their rafts to the assistance of the crew. Directly after the battle they indicate by a signal that they are able and ready to receive the sick and wounded." If the battle took place in Queenstown Harbour this might be possible, but in a naval battle in the open sea the raft brigade would hardly prove a success. As regards the plans for a hospital ship given by Dr. Braidwood, the amount of space allowed for service rooms seems too small, but their merits turn mainly on the arrangements which may be adopted for continuous forced ventilation, and these are not shown or referred to. It is, theoretically, possible to properly ventilate the wards which he indicates, but the practical difficulties in the way of doing this are considerable, and these must be satisfactorily overcome, or the ship may become a floating pest-house.

